# Clinical Applications of Flavonoids in Pregnancy: Potential Therapeutic Strategies for Gestational Disorders—An Update

**DOI:** 10.3390/nu18213482

**Published:** 2026-10-23

**Authors:** Aleksandra Kozłowska

**Affiliations:** Department of Social Medicine and Public Health, Medical University of Warsaw, Pawińskiego 3A Str., 02-106 Warsaw, Poland; aleksandra.kozlowska@wum.edu.pl

**Keywords:** flavonoids, pregnancy, pre-eclampsia, gestational diabetes, pregnancy outcomes, anthocyanins

## Abstract

Gestational disorders, including pre-eclampsia, gestational diabetes mellitus, and fetal growth restriction, represent major maternal–fetal health challenges, with limited pharmacological options that can be used safely throughout pregnancy. Flavonoids, a diverse group of plant-derived polyphenols, exert antioxidant, anti-inflammatory, and vasoprotective effects in preclinical models. Translational evidence from these mechanistic studies has increased interest in their potential role in maternal–fetal health. This narrative review evaluates the clinical evidence regarding maternal flavonoid exposure across preconception care, pregnancy complications, postpartum recovery, and offspring health. A comprehensive literature search identified thirteen studies published within the past five years, comprising randomized controlled trials, meta-analyses, and evidence from major prospective birth cohorts. Available clinical evidence suggests that higher dietary intake of flavonoid-rich fruits, berries, and vegetables is associated with a lower risk of gestational diabetes, gestational hypertension, and small-for-gestational-age births. Clinical studies of targeted supplementation with specific compounds, including quercetin, epigallocatechin gallate, and botanical extracts, have reported potential benefits in selected clinical settings, including endocrine and reproductive outcomes in women with polycystic ovary syndrome, blood pressure management in pre-eclampsia, and postpartum or post-cesarean pain. Furthermore, maternal flavonoid exposure has been associated with differences in offspring immune development and a potentially lower incidence of food allergies in early childhood. Overall, current clinical evidence supports further investigation of dietary flavonoid intake and selected flavonoid-based interventions in maternal–fetal medicine. Overall, while current evidence strongly supports habitual consumption of a varied, plant-rich diet naturally high in flavonoids during pregnancy, clinical data remain insufficient to recommend routine high-dose isolated flavonoid supplementation in standard obstetric practice.

## 1. Introduction

Pregnancy is a complex, dynamically regulated physiological state that requires substantial metabolic, immunological, and cardiovascular adaptations. These adaptive mechanisms are necessary to support both proper fetal development and maternal homeostasis [1]. Disruptions in early development can precipitate severe gestational complications. Among these, gestational diabetes mellitus (GDM), pre-eclampsia (PE), and fetal growth restriction (FGR) represent major global obstetric challenges [2,3,4]. These disorders are associated not only with perinatal morbidity and mortality but also with long-term health implications, including a significantly increased risk of developing cardiovascular disease, type 2 diabetes, and metabolic syndrome in both the mothers and their offspring later in life [5,6,7,8].

At the sub-cellular level, the pathophysiology of these gestational complications shares closely intertwined denominators, such as chronic low-grade inflammation, endothelial dysfunction, and excessive oxidative stress. Currently, the clinical management of gestational disorders remains a therapeutic dilemma. Due to the high susceptibility of the developing fetus to xenobiotic toxicity, the pharmacopoeia in obstetrics is highly restricted [9,10,11]. In recent years, natural polyphenolic compounds, such as flavonoids, have attracted attention as therapeutic and preventive agents in maternal–fetal medicine. A growing body of preclinical evidence indicates that flavonoids can modulate intracellular signaling cascades, which enhances the cellular antioxidant response and suppresses pro-inflammatory cytokine expression [12]. Furthermore, emerging data suggest that flavonoids can improve insulin sensitivity and enhance placental angiogenesis by regulating the balance of angiogenic factors [13,14,15].

Despite an expanding body of preclinical evidence demonstrating the therapeutic potential of flavonoids in gestational disorders, translated clinical research remains limited. Indeed, a search of the ClinicalTrials.gov database as of September 2026 yields only 25 registered clinical trials evaluating flavonoid safety, kinetics, or efficacy in pregnant cohorts. The aim of the present review is to provide an updated and comprehensive synthesis of the literature from 2022 to 2026 regarding the clinical applications of flavonoids in pregnancy. By contrasting mechanistic insights with human observational data, we aim to provide clinicians with a clear framework for navigating flavonoid use in obstetric practice.

## 2. Materials and Methods

This manuscript was designed as a structured narrative review informed by a systematic literature search strategy, aimed at evaluating the clinical efficacy, physiological mechanisms, and safety of flavonoids in maternal health and gestational outcomes.

A comprehensive literature search was conducted using the PubMed database to retrieve relevant clinical evidence investigating the therapeutic potential and clinical applications of flavonoids in pregnancy and gestational disorders. The search strategy combined Medical Subject Headings (MeSH) terms, Supplementary Concepts, and free-text keywords covering general terms and key individual compounds (e.g., flavonoids, anthocyanins, quercetin), combined with terms related to pregnancy (pregnancy, pregnancies). The following query in titles and abstracts were used: (“flavonoids” OR “flavonoid-rich” OR “anthocyanins” OR “flavonols” OR “flavones” OR “flavanones” OR “flavanols” OR “isoflavones” OR “quercetin” OR “kaempferol” OR “myricetin” OR “isorhamnetin” OR “luteolin” OR “apigenin” OR “hesperidin” OR “naringenin” OR “eriodictyol” OR “catechin” OR “epigallocatechin” OR “cyanidin” OR “delphinidin” OR “malvidin” OR “pelargonidin” OR “peonidin” OR “petunidin” OR “silymarin”) AND (“pregnancy” OR “pregnancy complications” OR “pregnancy outcomes” OR “gestational diabetes mellitus” OR “preeclampsia” OR “gestational hypertension” OR “delivery” OR “intrauterine growth restriction” OR “neonatal outcomes” OR “offspring outcomes” OR “fertility”). Filters were applied to restrict the search to the human clinical literature published within the last five years. The last search was performed on 1st of September 2026.

Studies were evaluated according to predefined eligibility criteria. The inclusion criteria comprised: (1) human studies, specifically randomized controlled trials (RCTs), non-randomized clinical trials, prospective observational cohort studies, and meta-analyses; (2) research evaluating the clinical effects, efficacy, or safety of flavonoids or flavonoid-rich interventions during pregnancy; (3) articles published in the English language; and (4) studies published within the recent 5-year timeframe. The exclusion criteria were as follows: (1) non-English publications; (2) case reports, editorials, commentaries, and secondary narrative reviews (excluded during primary record screening to prevent redundant data synthesis); and (3) preclinical investigations (in vitro cell studies or animal models) in the primary clinical literature synthesis. Literature screening was conducted in a two-step process. The initial database search yielded 27 records. Initial records were evaluated by title and abstract against the selection criteria, resulting in the exclusion of 14 records due to irrelevance. Subsequently, full-text manuscripts of potentially eligible publications were analyzed (4 RCTs, 7 prospective studies, and 2 meta-analyses). Reference management and citation tracking were performed using EndNote X7. Extracted data parameters included author details, study design, study population, specific flavonoid type and regimen (dosage and duration), target gestational condition, and main clinical outcomes. While the clinical review was restricted to recent human trials, no publication date restrictions were applied to complementary preclinical data. To offer a comprehensive perspective, the author incorporated a synthesis of the most up-to-date studies available, highlighting novel findings that may be less familiar to readers.

## 3. Dietary Sources, Intake, and Safety of Flavonoids in Pregnancy

Flavonoids are a major class of polyphenolic phytochemicals widely abundant in plant-derived foods [16,17]. They are broadly categorized into six primary subclasses based on chemical structure: flavonols, flavones, flavanones, flavanols, anthocyanins, and isoflavones [18]. Known for their potent antioxidant, anti-inflammatory, and metabolic-modulating properties, these bioactive compounds are considered to be potential protective agents in the prevention and treatment of chronic diseases [19,20,21,22].

Nevertheless, the systemic bioavailability of intact flavonoid aglycones is generally low. After ingestion, flavonoids undergo extensive intestinal and hepatic biotransformation involving both phase I and phase II reactions [23]. Metabolic transformation occurs in both the intestinal epithelium and the liver, with glucuronidation, sulfation, and methylation representing major conjugation pathways [24]. Concurrently, the gut microbiota metabolizes unabsorbed flavonoids into lower-molecular-weight phenolic metabolites [25]. Flavonoid metabolites are then eliminated through biliary and renal excretion, limiting systemic exposure to the parent compounds. Consequently, circulating concentrations of unchanged parent flavonoids in human plasma are generally low. Furthermore, inter-individual variability in flavonoid bioavailability reflects the combined influence of multiple factors. Relevant factors include the food matrix and processing, host metabolic capacity, genetic variation, dietary patterns, age, and sex. In addition, differences in gut microbial composition and metabolic activity may substantially contribute to inter-individual differences in systemic exposure [23].

Despite regional differences in dietary patterns, assessing population-level flavonoid intake remains challenging due to inconsistencies in dietary assessment methods and food composition databases. Consequently, national estimates vary substantially, ranging from 626 ± 579 mg/day among Australian adults to an average of 428 ± 49 mg/day across several European populations [26,27]. Lower intake levels have been reported in the United States, where the mean daily flavonoid consumption was estimated at 344.83 ± 9.13 mg/day [28]. Available dietary data suggest substantial variation in flavonoid intake during pregnancy across populations. In a Brazilian cohort of 785 pregnant women, the median daily intake of total flavonoids was 50 mg/day (interquartile range: 31–75 mg/day), which was characterized by the authors as very low [29]. In contrast, data from the U.S.-based CANDLE cohort indicated a considerably higher median flavonoid intake of approximately 288 mg/day during the second trimester [30]. These differences may reflect variation in dietary patterns, food sources, population characteristics, and methods used to assess flavonoid intake.

Interestingly, a growing body of evidence emphasizes the clinical importance of habitual dietary flavonoid intake during gestation. In a Brazilian cohort of pregnant women, higher intakes of total flavonoids [OR = 0.62; 95% CI: 0.38–0.96] and anthocyanidins [OR = 0.62; 95% CI: 0.40–0.99] were associated with lower odds of maternal obesity [29]. Furthermore, in another prospective cohort study, including 2231 pregnant women, higher dietary intakes of total polyphenols and flavonoids were associated with lower odds of gestational diabetes mellitus [OR = 0.55; 95% CI: 0.30–0.99, and OR = 0.57; 95% CI: 0.32–0.99, respectively] [31]. These findings suggest a potential association between higher consumption of plant-derived polyphenols and more favorable maternal metabolic outcomes. Consequently, public health and nutritional guidelines advocating increased dietary intake of fruits, vegetables, and minimally processed plant foods during pregnancy are strongly justified. Table 1 presents the content of specific flavonoids in food matrixes in relation to pregnancy-relevant dietary choices and safety profiles.

While flavonoids consumed as part of a balanced diet are generally considered safe, high-dose supplementation may pose risks of developmental toxicity [38]. Some flavonoids and their metabolites can cross the placental barrier. High pharmacological concentrations of these bioactive compounds may exert several toxicological effects: inhibition of folate and essential micronutrient transport, perturbation of drug-metabolizing enzymes and transporters, disruption of fetal cardiovascular homeostasis, and finally, phytoestrogenic endocrine activity [37,38,39,40]. Furthermore, excessive maternal intake of polyphenol-rich foods during the third trimester has been associated with premature constriction of the fetal ductus arteriosus (DA), increased pulmonary artery flow velocity, and right ventricular enlargement [41].

## 4. Preclinical Evidence on Flavonoids in Gestational Complications

Gestational complications, including pre-eclampsia and gestational diabetes mellitus, present major therapeutic challenges due to strict safety limitations during pregnancy. Both conditions stem from low-grade chronic inflammation and excessive oxidative stress, which together impair endothelial function, compromise placental perfusion, and drive trophoblast apoptosis. Beyond their benefits in these primary metabolic and vascular disorders, flavonoids also show therapeutic potential for managing secondary pregnancy-related complications. In particular, their broad-spectrum biological activity addresses infectious diseases, environmental toxicant exposure, neurodevelopmental abnormalities, and reproductive dysfunction. This subsection details the specific signaling pathways and molecular mechanisms through which flavonoids exert their potential protective effects across experimental models (Table 2 and Table 3).

### 4.1. Preclinical Evidence for the Protective Effects of Flavonoids in Pre-Eclampsia

Pre-eclampsia (PE) is a multifactorial pregnancy disorder characterized by placental ischemia, exaggerated systemic inflammation, oxidative stress, and widespread endothelial dysfunction. Increasing evidence from experimental studies suggests that flavonoids such as baicalin, kaempferol, luteolin, and silibinin may exert protective effects against the development and progression of PE.

Among the flavonoids investigated, baicalin has demonstrated significant endothelial protective properties [42]. Cao et al. evaluated the effect of baicalin on human umbilical vein endothelial cells (HUVECs) exposed to hydrogen peroxide (H_2_O_2_), a commonly used in vitro model of pre-eclamptic oxidative stress, and showed that baicalin dose-dependently inhibited the expression and phosphorylation of the mitogen-activated protein kinase extracellular signal-regulated kinase 1/2 (MAPK/ERK1/2) signaling pathway. These effects were at least partly mediated by a reduction in endothelial cell apoptosis and restoration of myeloperoxidase (MPO) secretion through a tumor necrosis factor alpha (TNF-α)-dependent mechanism. This study suggested that baicalin plays an important role in maintaining endothelial integrity under oxidative conditions.

Another flavonoid, kaempferol, has also shown promising therapeutic potential in experimental models of PE [43]. Its efficacy was evaluated in L-NAME-induced pre-eclamptic rats and cellular hypoxia/reoxygenation models. In these experimental systems, kaempferol attenuated maternal hypertension and proteinuria, mitigated fetal growth restriction, and reduced tissue oxidative stress. At the cellular level, these beneficial outcomes correlated with the restoration of autophagic flux and enhanced mitophagy, marked by upregulated mitophagy-related proteins PINK1 and Parkin expression, suggesting improved mitochondrial quality control and maintenance of cellular homeostasis. Furthermore, kaempferol promoted signal transducer and activator of transcription 3 (STAT3) phosphorylation and its mitochondrial translocation, suggesting enhanced mitochondrial and cellular protection, while suppressing NOD-like receptor family pyrin domain containing 3 (NLRP3) inflammasome activation and pyroptotic cell death, indicating reduced inflammatory responses. Together, these mechanisms contributed to the structural and functional recovery of placental and vascular tissues.

Similarly, luteolin has demonstrated protective effects in a reduced uterine perfusion pressure (RUPP) model of placental ischemia [44]. Treatment with luteolin (20 mg/kg/day) significantly reduced mean arterial pressure (MAP), uterine artery resistance index (UARI), and circulating levels of soluble fms-like tyrosine kinase-1 (sFlt-1) and endothelin-1 (ET-1), two key mediators implicated in endothelial dysfunction and hypertension during PE. Notably, these improvements occurred without adverse maternal or fetal outcomes, highlighting this flavonoid’s favorable safety profile in experimental settings.

Another flavonoid with demonstrated efficacy in PE is silibinin [45]. In L-NAME-induced pre-eclamptic rats, oral silibinin administration (100 mg/kg/day, on days 0, 7, and 14 of pregnancy) significantly decreased systolic blood pressure and 24-h proteinuria. Furthermore, tissue analyses of the placenta and liver revealed marked reductions in key pro-inflammatory cytokines, including TNF-α, interleukin 1β (IL-1β), and interferon gamma (IFN-γ). Silibinin treatment also downregulated heat shock protein 70 (Hsp70) expression and inhibited nuclear factor kappa B (NF-κB) activity, indicating that its protective benefits are mediated, at least in part, through the suppression of inflammatory signaling pathways.

Collectively, these preclinical findings suggest that flavonoids may represent promising candidates for the prevention and management of PE by targeting multiple pathogenic mechanisms, including oxidative stress, inflammation, endothelial dysfunction, impaired autophagy, and abnormal placental signaling.

### 4.2. Preclinical Evidence for the Protective Effects of Flavonoids in Gestational Diabetes Mellitus

Gestational diabetes mellitus is characterized by impaired glucose tolerance, severe insulin resistance, chronic low-grade inflammation, oxidative stress, and placental metabolic dysfunction. Recent experimental studies have highlighted the potential of flavonoids, especially anthocyanin-rich plant extracts, to improve glycemic control, lipid metabolism, inflammatory status, and pregnancy outcomes in GDM.

Numerous studies have reported anti-gestational diabetic effects of flavonoids in animal and cell-culture studies. Homoplantaginin, a flavonoid from the flavone subclass, has been shown to exert metabolic effects in experimental GDM models [46]. Administration of this substance significantly reduced fasting blood glucose levels and attenuated both oxidative stress and inflammatory responses in GDM rats after 2 weeks of treatment. Interestingly, these improvements were accompanied by enhanced offspring survival, suggesting beneficial effects on both maternal and fetal health.

Tectorigenin, a flavonoid from the isoflavone subclass, was also shown to possess anti-inflammatory activity in streptozotocin (STZ)-induced GDM rats [47]. Oral treatment markedly reduced blood glucose levels, insulin resistance, circulating free fatty acids, visfatin, and markers of lipid peroxidation. In addition, significant improvements in lipid profiles were also observed. Tectorigenin also suppressed the production of numerous inflammatory mediators and signaling molecules, including TNF-α, IL-1β, IL-6, transforming growth factor beta (TGF-β), monocyte chemoattractant protein-1 (MCP-1), intercellular adhesion molecule-1 (ICAM-1), vascular cell adhesion molecule-1 (VCAM-1), NF-κB, cyclooxygenase-2 (COX-2), inducible nitric oxide synthase (iNOS), toll-like receptor 4 (TLR4), myeloid differentiation primary response 88 (MyD88), and NLRP3. Simultaneously, it enhanced insulin secretion, improved homeostatic model assessment of insulin resistance (HOMA-β) and homeostatic model assessment of insulin sensitivity index (HOMA-ISI) indices, increased hepatic glycogen storage, and elevated fetal and placental weights. These findings indicate that tectorigenin improves metabolic homeostasis through coordinated regulation of oxidative stress and inflammatory signaling pathways.

In an (STZ)-induced model of gestational diabetes mellitus in female rats, daily oral administration of alpinumisoflavone (5, 10, and 20 mg/kg) for 20 days ameliorated hyperglycemia, HbA1c concentrations, and responses to oral glucose and insulin tolerance tests, as compared to the control animals [48]. Interestingly, alpinumisoflavone also attenuated oxidative stress and inflammatory responses by reducing the expression of COX-2, ICAM-1, VCAM-1, TLR4, MyD88, NF-κB p65, and NLRP3. Thus, the observed effects appear to be largely mediated through inhibition of the TLR4/MyD88/NF-κB signaling pathway.

Yang et al. investigated the metabolic effect of icariin in trophoblast cell models exposed to high glucose concentrations [49]. In HTR-8/SVneo trophoblasts, icariin protected against glucose-induced cellular injury and apoptosis while reducing intracellular lactate dehydrogenase (LDH) levels. Further analyses revealed that these effects were mediated through modulation of the S100 calcium-binding protein A9 (S100A9)/receptor for advanced glycation end products (RAGE) signaling pathway, resulting in enhanced mitophagy and improved cellular survival. Baicalin has also demonstrated beneficial effects in GDM animal models, where it improved lipid metabolism and immune function through inhibition of the Ras homolog family member A (RhoA)/Rho-associated coiled-coil-containing protein kinase (ROCK) signaling pathway [50]. These findings further support the broad spectrum of biological activities exhibited by flavonoids in metabolic disorders associated with pregnancy.

In addition to isolated flavonoids, anthocyanin-rich extracts were investigated in STZ-induced gestational diabetic rats [51]. An anthocyanin-rich bilberry (*Vaccinium myrtillus* L.) extract treatment (400 mg/kg/day, starting 6 weeks before mating and continuing throughout the pregnancy) by gavage significantly alleviated hyperglycemia, insulin resistance, dyslipidemia, oxidative stress, and inflammation in GDM rats. Notably, these improvements were associated with increased abundance of beneficial *Lactobacillus* species and modulation of the gut microbiota–bile acid–Takeda G protein-coupled receptor 5 (TGR5)/farnesoid X receptor (FXR)–glucagon-like peptide-1 (GLP-1) axis. These findings suggest that interactions between dietary polyphenols and the gut microbiota may represent an important mechanism underlying the metabolic benefits of flavonoid-rich interventions during pregnancy.

Altogether, accumulating evidence indicates that flavonoids exert potential multifaceted protective effects in GDM by improving glucose and lipid metabolism, enhancing antioxidant defenses, suppressing inflammatory signaling, and preserving placental function. Although most findings currently originate from experimental studies, they provide a strong rationale for further investigation of the effectiveness of flavonoids in the prevention or management of GDM.

### 4.3. Flavonoids in Maternal–Fetal Infectious Complications

Several flavonoids have shown promising activity against pathogens that can compromise pregnancy outcomes and fetal development. Listeria monocytogenes (LM) infection during early pregnancy induces embryonic loss by triggering NLRP3 inflammasome activation and pyroptosis at the maternal–fetal interface. In a pregnant BALB/c mouse model of LM-induced fetal loss, administration of baicalin (BA; administered from GD 0.5 to GD 6.5) significantly reduced embryonic resorption and preserved pregnancy outcomes [52]. BA treatment markedly suppressed NLRP3 and apoptosis-associated speck-like protein containing a caspase recruitment domain (ASC) protein expression, attenuated caspase-1 activation and gasdermin D (GSDMD) cleavage, and decreased IL-1β release in uterine tissues. Further in vitro analyses in macrophages demonstrated that baicalin (BA) inhibited LM-induced NLRP3 inflammasome assembly by disrupting NIMA-related kinase 7 (NEK7)–NLRP3 interactions and downregulating c-Jun *N*-terminal kinase (JNK) phosphorylation without altering total NEK7 levels. Overall, BA mitigates LM-induced embryonic loss by suppressing uterine pyroptosis and inflammasome activation via modulation of the JNK/NEK7–NLRP3/GSDMD signaling axis.

Similarly, regarding protozoan-induced intrauterine complications, quercetin has exhibited potent antiparasitic activity against *Toxoplasma gondii*, one of the most clinically significant congenital pathogens [53]. In human placental villous explants, quercetin inhibited parasite adhesion, invasion, and intracellular proliferation. Furthermore, treatment reduced lipid droplet accumulation within infected tissues, restored redox homeostasis, and modulated inflammatory cytokine production, including IL-4, IL-6, and IL-8. In silico analyses additionally identified the parasite enzyme hypoxanthine-guanine phosphoribosyltransferase (*TgHGPRT*) as a potential molecular target of quercetin. Beyond bacterial and protozoan models, flavonoids’ broad-spectrum antimicrobial activity extends to viral infections during pregnancy. In this context, naringenin has attracted attention for its combined antiviral and anti-inflammatory properties [54]. In both human placental explants and pregnant mouse models of Zika virus (ZIKV) infection, naringenin significantly reduced viral replication and attenuated inflammatory responses. Notably, the compound counteracted lipopolysaccharide (LPS)-induced exacerbation of viral infection and mitigated neonatal brain abnormalities associated with congenital ZIKV exposure, including structural alterations linked to microcephaly.

### 4.4. Flavonoids in Environmental Toxicant-Induced Pregnancy Complications

Environmental toxicants represent an increasingly recognized threat to maternal and fetal health. To evaluate the protective efficacy of fisetin against methylmercury (MeHg)-induced developmental toxicity, pregnant Wistar rats (GD 0) were stratified into six experimental groups: control, fisetin alone (20 mg/kg), two doses of MeHg (0.5 and 2.5 mg/kg), and their respective co-treatments with fisetin. Interventions were administered orally on gestational days 7, 12, and 17 [55]. In rat offspring exposed prenatally to MeHg, supplementation of fisetin attenuated nephrotoxicity and renal fibrosis, normalized blood urea nitrogen (BUN) and creatinine concentrations, and reduced the expression of profibrotic markers, indicating preservation of renal structure and function.

Similarly, quercetin has shown efficacy against placental toxicity induced by zinc oxide nanoparticles (ZnO NPs) [56]. In pregnant rats, quercetin treatment (50 mg/kg) reduced oxidative stress, suppressed endoplasmic reticulum (ER) stress signaling, and inhibited apoptosis within placental tissues. These findings suggest that quercetin may protect placental integrity against nanoparticle-mediated cellular injury and dysfunction.

### 4.5. Flavonoids in Offspring Development and Reproductive Health

Beyond direct maternal benefits, several flavonoids have demonstrated the ability to improve neurodevelopmental and reproductive outcomes in offspring. Recently, it was reported that chrysin, a flavone from bee products and plants, effectively attenuated social deficits, anxiety-like behaviors, and hyperalgesia in a valproic acid (VPA)-induced model of autism spectrum disorder (ASD) in juvenile rats [57]. Postnatal supplementation with chrysin (100 mg/kg/day p.o. from PND 21 to 42) significantly ameliorated behavioral abnormalities in the ASD model in male offspring. These improvements were associated with modulation of corticohippocampal histone deacetylase (HDAC) activity and regulation of SH3 and multiple ankyrin repeat domains protein 3 (SHANK3) expression. Therefore, chrysin’s neuroprotective effects against ASD may be mediated through epigenetic mechanisms.

Anthocyanin-rich cherry juice has also shown considerable neuroprotective potential. In rat offspring exposed to maternal immune activation induced by lipopolysaccharide, supplementation of anthocyanin-rich extract improved both short- and long-term memory performance, reduced anxiety-related behaviors, and restored normal hippocampal cell numbers [58]. Hence, these findings indicate that anthocyanins may mitigate neuroinflammation and preserve cognitive function during early development.

In addition to neuroprotection, flavonoids may directly support placental health and trophoblast function. Pre-treatment of HTR-8/SVneo trophoblast cells exposed to oxidative stress with quercetin enhanced cell viability and migratory capacity and reduced apoptosis [59]. Quercetin also exerted these effects through activation of the epidermal growth factor receptor (EGFR)/phosphoinositide 3-kinase (PI3K)/protein kinase B (AKT)–nuclear factor erythroid 2-related factor 2 (NRF2)/heme oxygenase-1 (HO-1) signaling pathway, which may contribute to the maintenance of trophoblast integrity. Given that oxidative stress-induced trophoblast dysfunction represents a primary pathological mechanism in recurrent spontaneous abortion, these findings highlight the therapeutic potential of quercetin in early pregnancy maintenance.

Similarly, promising effects of baicalein were reported in a study that used a recurrent pregnancy loss (RPL) mouse model [60]. Treatment with baicalein (10 or 40 mg/kg, E0.5–E12.5) significantly attenuated placental injury and improved pregnancy outcomes. Baicalein effectively inhibited ferroptosis by limiting iron accumulation and lipid peroxidation. These effects were mediated through activation of the Nrf2/GPx4 signaling axis, a key regulatory pathway involved in cellular antioxidant defense and ferroptosis suppression.

Evidence of reproductive benefits has also been reported in veterinary models [61]. A natural antioxidant blend consisting of tocopherol and rosemary-derived flavonoid extract enhanced maternal antioxidant defenses during the periparturient period in Beetal goats. Supplementation improved the biochemical composition of colostrum and milk while increasing neonatal birth weight, suggesting beneficial effects on both maternal physiology and offspring development.

## 5. Recent Clinical Evidence on Flavonoid Exposure and Interventions During Pregnancy: A Five-Year Update

Flavonoids have been thoroughly examined in preclinical research as bioactive substances having advantageous effects during gestation. Nevertheless, clinical evidence about flavonoid consumption or supplementation during gestation remains far more limited. Because of safety considerations and ethical constraints, research in this population relies heavily on prospective observational cohort studies, alongside a growing but still limited number of randomized controlled trials and meta-analyses (Supplementary Table S1). This update highlights findings from recent clinical and prospective studies that provide compelling evidence regarding the impact of maternal flavonoid exposure on preconception health, fertility, maternal metabolic status, gestational outcomes, and offspring development.

### 5.1. Flavonoids in Pre-Conception and Fertility Outcomes

Despite extensive research on the metabolic benefits of flavonoids, clinical trials evaluating their supplementation during the preconception period and in female subfertility remain scarce. Preconception care represents a critical window where systemic low-grade inflammation, intrafollicular oxidative stress, and endocrine dysregulation—most notably in polycystic ovary syndrome (PCOS)—impair folliculogenesis, oocyte quality, and implantation success [62,63,64]. Vaez et al. evaluated the efficacy of quercetin supplementation (500 mg/day for 40 days) in 64 infertile women with PCOS undergoing in vitro fertilization (IVF) [65]. Dietary quercetin significantly improved endocrine and inflammatory profiles by markedly reducing serum levels of luteinizing hormone (LH), IL-6, TNF-α, and reactive oxygen species (ROS) compared with placebo. Additionally, these systemic improvements translated directly into superior reproductive endpoints. The quercetin arm yielded a higher proportion of mature Metaphase II oocytes (79.5% vs. 63.6%; *p* = 0.03), better embryo quality (Grade AB: 67.1% vs. 63.2%), and a significantly elevated clinical pregnancy rate (83.9% vs. 12.1%). These results further demonstrate quercetin’s ability to suppress intra-ovarian inflammatory signaling, attenuate microenvironmental oxidative stress, and downregulate LH-driven androgen biosynthesis. However, given the small cohort size and the unusually large difference in pregnancy rates, these promising results should be interpreted with caution and require confirmation in larger prospective trials [65].

### 5.2. Gestational Disorders and Complications

Gestational disorders, including gestational diabetes mellitus, gestational hypertension (GH), pre-eclampsia, and fetal growth restriction (FGR), involve overlapping pathophysiological pathways characterized by chronic low-grade inflammation, increased oxidative stress, placental vascular dysfunction, and metabolic dysregulation [66,67,68]. Given their systemic antioxidant, anti-inflammatory, and vasoprotective properties, dietary flavonoids and polyphenol-rich interventions may represent a promising non-pharmacological strategy to mitigate maternal and fetal complications during pregnancy [69,70,71].

#### 5.2.1. Gestational Diabetes Mellitus

Large-scale observational cohorts and dietary RCTs support the protective role of dietary flavonoids against GDM, whereas isolated pill-based supplementation shows more nuanced outcomes. In a prospective birth cohort including 93,759 pregnant women (Japan Environment and Children’s Study; 2096 GDM cases, 2.2%), Kojima et al. evaluated first-trimester dietary intake using a validated food frequency questionnaire (FFQ) [72]. The authors identified a statistically significant inverse association between GDM risk and maternal intake of total fruit (aOR = 0.82), flavonoid-rich fruits (aOR = 0.80), and 100% fruit juice (aOR = 0.91 per 100 mL/day), whereas non-flavonoid fruits showed no significant association. These epidemiological findings are reflected in clinical study, reinforced by Jaworsky et al. [73]. In a 12-week intervention among 38 women with GDM, a daily dietary intake of 1 cup of whole berries (rich in anthocyanins and flavonols) and 1 cup of leafy vegetables, combined with postprandial walking, significantly improved blood glucose, elevated HDL cholesterol, enhanced serum antioxidant capacity, and reduced pro-inflammatory interleukin-6 (IL-6; *p* < 0.05) levels. Conversely, Soldavini et al. evaluated a 12-week treatment with a purified supplement containing anthocyanins (108 mg/day), omega-3 fatty acids (2.4 g/day), and alpha-cyclodextrins in 40 women with GDM at 24–28 weeks of gestation. The intervention was compared with individualized one-to-one dietary counseling alone [74]. Interestingly, the supplement intake produced no statistically significant additive changes in metabolic or inflammatory markers over dietary counseling. These data suggest that structured dietary modifications provide a primary baseline effect that may overshadow low-dose isolated flavonoid extracts in small clinical samples. Importantly, translating these findings requires methodological caution due to multi-component study designs. In the dietary trial, concurrent postprandial walking prevents isolating the independent metabolic effects of flavonoids from exercise-induced glucose uptake. Similarly, the supplement trial evaluated a combination of anthocyanins, omega-3 fatty acids, and alpha-cyclodextrins, making it impossible to attribute the outcomes solely to flavonoid monotherapy.

#### 5.2.2. Hypertensive Disorders of Pregnancy

Maternal vascular adaptation during early gestation is heavily influenced by systemic inflammatory tone, where flavonoids exert notable vasoprotective effects [75]. In a prospective cohort of 2160 pregnant women, Liu et al. found that higher early-pregnancy intake of dietary polyphenols and flavonoids, predominantly from fruit, was associated with a lower risk of gestational hypertension [76]. Notably, multivariable logistic regression analyses demonstrated nonlinear inverse associations, with risk reductions plateauing at approximately 450 mg/day for total polyphenols, 300 mg/day for flavonoids, and 80 mg/day for phenolic acids. Importantly, mediation analysis suggested that modulation of systemic inflammation, assessed using the systemic immune-inflammation index, accounted for 8.04% of the association between flavonoid intake and GH risk and 8.19% of that for total polyphenol intake. Extending these observations to clinical intervention settings, a meta-analysis of 14 RCTs by Nguyen et al. evaluated polyphenol candidates co-administered with standard antihypertensive therapy (nifedipine) in the acute management of pre-eclampsia [77]. Adjuvant administration of epigallocatechin gallate (EGCG) or resveratrol significantly reduced the time to blood pressure control (mean difference MD = −14.10 min for EGCG; −15.50 min for resveratrol) and extended the interval to the next hypertensive crisis (MD = +3.10 h for EGCG; +2.50 h for resveratrol) compared with nifedipine monotherapy, highlighting EGCG’s potent synergistic effect on vascular smooth muscle relaxation and endothelial nitric oxide pathways. However, these promising trial findings must be interpreted with caution. The ~14–16 min reduction in acute blood pressure control represents a surrogate endpoint of modest clinical relevance. Furthermore, as EGCG is a flavanol and resveratrol a non-flavonoid stilbene, these results cannot be generalized across all flavonoid subclasses. Given the small cohort sizes and absent fetal hemodynamic monitoring, current evidence remains insufficient to support routine adjunctive polyphenol therapy in severe pre-eclampsia.

#### 5.2.3. Fetal Growth and Neonatal Outcomes

Beyond maternal metabolic parameters, maternal flavonoid consumption has been associated with fetal development and birth outcomes. In a community-based cohort study of 2244 pregnant women, including 234 cases of small-for-gestational-age (SGA), Zhang et al. assessed dietary anthocyanidin intake, with a median intake of 28.7 mg/day, predominantly comprising cyanidin and peonidin [78]. Higher anthocyanidin intake was significantly associated with a lower risk of SGA (OR = 0.96), with the inverse association being more pronounced among women consuming diets with a higher inflammatory potential. These findings suggest that higher anthocyanidin intake may partly mitigate the adverse association between a pro-inflammatory dietary pattern and fetal growth. Extending these findings beyond birth outcomes, an observational twin cohort study of 143 twin families further highlighted the potential long-term relevance of early-life metabolic pathways [79]. The study revealed that preterm birth was associated with alterations in the gut microbiota of infants at 12 months of age. Untargeted metabolomics identified 18 metabolites associated with physical and neurobehavioral development, among which catechin was linked to flavonoid and phenylpropanoid biosynthesis. These findings suggest that alterations in flavonoid-related metabolic pathways may be relevant to postnatal developmental trajectories [79].

### 5.3. Flavonoids in Postpartum and Perioperative Care

Effective pain management during the postpartum and perioperative periods is critical for maternal recovery, early mobilization, and the establishment of mother–infant bonding [80]. Conventional analgesic strategies relying on nonsteroidal anti-inflammatory drugs (NSAIDs) or opioids are frequently constrained by adverse effects—such as gastrointestinal distress, nausea, vomiting, or sedation—prompting increased interest in safe and effective adjunctive modalities [81,82].

In perioperative obstetric care, preoperative administration of pure flavonoids has shown promising analgesic effects in women undergoing cesarean delivery [83]. In a double-blind randomized controlled trial involving 80 patients undergoing elective cesarean section under spinal anesthesia, a single oral dose of 500 mg quercetin administered 1 h before surgery significantly reduced Visual Analog Scale (VAS) pain scores at all evaluated time points (2, 6, 12, and 24 h postoperatively; *p* < 0.001). Furthermore, quercetin significantly prolonged the time to the first rescue analgesic request (3.9 ± 1.3 h vs. 2.73 ± 0.78 h in the placebo group; *p* < 0.001) and accelerated the initiation of physical activity (15.2 ± 1.9 h vs. 19.03 ± 2.66 h; *p* < 0.001). Although cumulative 24-h morphine consumption did not differ significantly between groups (58.3 ± 6.48 mg vs. 58.9 ± 6.2 mg; *p* = 0.673), patients receiving quercetin reported significantly higher satisfaction scores on the second postoperative day (*p* = 0.042). These effects may be related to quercetin’s anti-inflammatory and antioxidant properties, including its potential to attenuate pro-inflammatory signaling and oxidative stress. However, the underlying mechanisms were not directly assessed in this clinical trial.

Regarding postpartum care following normal vaginal delivery, significant benefits have been demonstrated in mitigating painful uterine contractions (afterpains) using *Elaeagnus angustifolia* (Russian olive/Senjed) fruit extract, a rich source of bioactive flavonoids including catechin, epicatechin, quercetin, and kaempferol [84]. A triple-blind, three-arm randomized controlled trial in 102 multiparous women revealed that oral administration of 500 mg *E. angustifolia* extract every 6 h for 24 h (initiated 2 h postpartum) markedly reduced both the intensity and sensory/emotional quality of afterpains. One hour following the fourth dose, the mean Postpartum Pain Observation Measure Visual Analog Scale (POM-VAS) score was 0.9 ± 1.6 in the extract group compared to 2.0 ± 1.7 in the placebo group and 1.7 ± 1.0 in the control group (*p* = 0.004). Crucially, this intervention led to a dramatic reduction in rescue analgesic consumption (mefenamic acid), dropping from 2.4–2.5 capsules in the reference groups to 0.41 ± 0.7 capsules in the intervention arm (*p* < 0.001). These analgesic effects may be related to the anti-inflammatory properties of flavonoids present in *E. angustifolia*, including their potential inhibition of cyclooxygenase and nitric oxide synthase pathways and consequent reduction in prostaglandin and nitric oxide signaling.

### 5.4. Flavonoids and Offspring Health Outcomes

Maternal nutrition during pregnancy plays an important role in fetal immune programming, gut colonization, and long-term pediatric susceptibility to allergic and immune-mediated diseases [85,86]. Beyond immediate gestational effects, prenatal maternal intake of bioactive flavonoids—specifically isoflavones and proanthocyanidins—has been increasingly linked to a reduced risk of offspring food allergies during infancy and early childhood through the modulation of maternal–fetal immune tolerance and gut-microbiota-derived metabolite axes [87,88].

In a large prospective birth cohort from the Japan Environment and Children’s Study comprising up to 86,012 mother–child pairs, Yang et al. investigated the association between maternal dietary isoflavone intake during pregnancy, estimated as the combined intake of daidzein and genistein, and physician-diagnosed food allergy in offspring up to 3 years of age [87]. Higher maternal isoflavone intake was significantly inversely associated with the risk of childhood food allergy. Notably, sex-specific differences and distinct age-related patterns were observed. The inverse association was consistently observed across both age groups (≤1 year and 1–3 years) among female offspring, whereas among male offspring, a significant inverse association was observed only in children aged 1–3 years. However, the causal nature of this association remains unclear, highlighting the need for further interventional studies to establish causality.

Complementing these epidemiological findings, Dawson et al. investigated the association between maternal prenatal proanthocyanidin intake and infant food allergy in a prospective prebirth cohort of 599 mother–infant dyads [88]. Higher maternal dietary proanthocyanidin intake was significantly associated with a lower risk of infant food allergy at 12 months of age (OR = 0.58; 95% CI 0.36–0.96). Importantly, targeted metabolomic and microbiome profiling identified maternal urinary hippuric acid, an end-product of dietary polyphenol metabolism, as a potential metabolic correlate of this association; higher urinary hippuric acid concentrations were independently associated with a lower risk of infant food allergy (OR = 0.62; 95% CI 0.42–0.93). Further integrated host–microbiome analyses showed that maternal carriage of *Prevotella copri* during the third trimester was associated with 21% higher urinary hippuric acid concentrations. However, no evidence of mediation was observed. Together, these findings highlight a potential maternal diet–gut microbiota–polyphenol metabolite axis that may contribute to early-life immune development and susceptibility to food allergy [88].

## 6. Summary

While preclinical studies provide evidence for the multi-target actions of flavonoids, clinical translation in human pregnancy remains strictly constrained. The present narrative review synthesizes current evidence on the effects of maternal flavonoid intake during pregnancy. Current clinical data rely mostly on prospective observational cohort studies, which inherently restrict the ability to establish definitive cause-and-effect relationships due to potential residual confounding, self-reported dietary assessments, and matrix effects of whole-food diets. Nevertheless, clinical data suggest that maternal flavonoid exposure—whether through standardized dietary patterns or target compound administration—exerts protective effects across preconception health, maternal metabolic regulation, acute vascular adaptation, and long-term offspring immune programming.

In clinical practice, maternal dietary intake of flavonoid-rich foods represents a low-risk, highly accessible non-pharmacological strategy to mitigate common gestational disorders. Over the past five years, prospective cohort data and dietary interventions demonstrate that early-trimester consumption of whole flavonoid-rich fruits, berries, and vegetables is associated with a reduced risk of gestational diabetes mellitus, lower rates of small-for-gestational-age births, and attenuation of gestational hypertension. Furthermore, prenatal exposure to specific subclasses such as isoflavones and proanthocyanidins has been associated with differences in offspring immune outcomes, while casuality remains uncertain.

Targeted supplementation with purified flavonoids or standardized botanical extracts has shown therapeutic potential in specific clinical scenarios. In preconception care for infertile women with PCOS, oral quercetin markedly improves endocrine parameters, reduces intrafollicular oxidative stress, and enhances clinical pregnancy rates following IVF. In acute pre-eclampsia management, adjuvant administration of EGCG or resveratrol synergizes with conventional antihypertensives to accelerate blood pressure stabilization. Moreover, perioperative and postpartum application of pure quercetin or *Elaeagnus angustifolia* extract significantly alleviates post-cesarean pain and uterine afterpains, drastically reducing the requirement for rescue analgesics.

Although maternal flavonoid exposure and targeted supplementation show promising safety and efficacy profiles, several methodological limitations must be considered when interpreting these findings. To avoid inappropriate clinical translation, evidence regarding gestational flavonoid exposure must be interpreted within three distinct exposure tiers: (1) habitual dietary intake via whole-food matrices (fruits, vegetables, berries, tea), which represents low-dose exposure supported by observational cohorts; (2) whole-food interventional patterns; and (3) isolated high-dose supplementation (e.g., purified quercetin, EGCG isolates, or concentrated botanical extracts). Conclusions derived from observational dietary cohorts reflect broad lifestyle patterns and cannot be extrapolated to predict the efficacy or safety of isolated pharmacological supplementation. First, the limited number of clinical studies investigating flavonoid interventions in pregnant women, particularly prospective studies, restricts the available evidence and limits the generalizability of the findings. Second, as this was a narrative review, data extraction and interpretation were not independently performed by multiple reviewers, which may have introduced selection and interpretation bias. Moreover, literature retrieval was restricted to PubMed/MEDLINE; although PubMed indexes the overwhelming majority of the peer-reviewed clinical obstetric literature, relevant studies indexed exclusively in Embase, Scopus, or Cochrane CENTRAL may not have been captured. Further the current clinical evidence is the small sample size and short duration of randomized controlled trials, alongside marked heterogeneity in compound types, dosages, and administration matrices. Furthermore, prospective cohort studies relying on dietary questionnaires are subject to recall bias, potential underreporting, and residual confounding from co-consumed polyphenol-rich foods. Additionally, many interventional designs struggle to isolate independent flavonoid effects from concurrent dietary or lifestyle modifications. This review does not provide personalized or trimester-specific recommendations. Potential biases regarding geographic population homogeneity, single-point biomarker assessments, and limited long-term pediatric follow-up further complicate the translation of these results into standardized clinical guidelines. Therefore, these methodological variations emphasize the necessity for well-powered, standardized multi-center trials in future maternal–fetal research.

Clinical Key Message: In conclusion, clinicians should actively encourage pregnant women and those planning pregnancy to adopt a diet naturally rich in diverse flavonoids (such as berries, dark leafy greens, and citrus fruits) as a safe baseline intervention to support metabolic homeostasis and fetal development. However, routine administration of high-dose, isolated flavonoid supplements during pregnancy cannot be broadly recommended at present. Supplementation should be reserved for specific, evidence-backed adjunctive indications (e.g., preconception PCOS management or perioperative pain protocols) and conducted under strict supervision. To bridge current clinical gaps, future multicenter RCTs must prioritize four core research directions: (1) establishing standardized dose-response matrices for purified isolates versus whole-food matrices; (2) characterizing trimester-specific maternal–fetal pharmacokinetics and placental transfer dynamics; (3) determining optimal timing of intervention across gestational windows (e.g., preconception vs. early vs. late trimester); and (4) implementing long-term pediatric follow-up protocols to evaluate offspring metabolic, cardiovascular, and neurodevelopmental trajectories. Furthermore, while habitual dietary flavonoid intake within a whole-food matrix is well-established as safe, dedicated clinical safety data on high-dose isolated supplementation in human pregnancy remain sparse and reliant on limited historical reporting; thus, cautious translation of preclinical toxicological models is required, underscoring the need for specialized maternal–fetal safety studies.

## Figures and Tables

**Table 1:** Content of flavonoids in chosen foodstuffs (mg/100 g foodstuff) in relation to pregnancy-relevant dietary choices and safety profiles; authors’ selection based on [32,33,34,35,36,37].

Flavonoids (mg/100 g)					
Fruits		Vegetables		Spices and Herbs	
Elderberries ^1^	518.05	Cabbage, red	210.16	Parsley, dried ^6^	4854.49
Chokeberries	368.66	Dock ^4^	102.20	Oregano, Mexican, dried	1545.79
Currants, black	170.41	Kale	92.98	Capers, raw	493.03
Blueberries	180.82	Fennel ^5^	84.50	Parsley, fresh	233.16
Blackberries	147.63	Radishes	63.99	Peppermint, fresh	60.48
Cranberries	99.22	Onions, red	56.61	Thyme, fresh	47.75
Currants, red	79.49	Arugula	47.11	Nuts	
Kumquats	79.26	Chard	25.60	Pecans	34.01
Raspberries	55.57	Celery hearts, green	22.60	Pistachios	14.37
Grapefruits ^2^	55.40	Artichokes	22.28	Hazelnuts	11.96
Lemons	53.38	Peppers, hot chili	21.17	Almonds	11.00
Grapes, red ^3^	52.42	Broad beans, cooked	20.63	Beverages	
Limes	46.80	Chives	17.11	Black tea, brewed ^7^	118.35
Oranges	43.49	Asparagus, cooked	15.16	Black currant juice	78.04
Cherries	40.00	Cress, fresh	14.00	White tea, brewed ^8^	74.60
Strawberries	33.52	Chicory, green	11.79	Green tea, brewed ^9^	53.84
Plums	14.42	Lettuce, red	11.72	Oolong tea, brewed ^10^	52.37
Apples	13.73	Spinach	11.44		
Apricots	10.67	Endive	10.10	Orange juice	24.13
Pears	8.01	Brussels sprouts, cooked	7.68	Pink grapefruit juice ^11^	17.97
Bananas	6.21	Peppers, green	6.98		
Legumes		Sweets		Cereals and grains	
Cowpeas, black	277.41	Dark chocolate ^12^	108.60	Wheat, purple	25.85
Beans, black	28.00	Cocoa, dry powder ^13^	106.68	Buckwheat	15.38
Beans, kidney, red	10.87	Milk chocolate ^14^	15.04	Sorghum, red	8.43

^1^ Rich in anthocyanins. Must be fully cooked; raw elderberries contain cyanogenic glycosides and should be avoided. ^2 and 11^ Note: Check for drug interactions if on antihypertensives. ^3^ Contains resveratrol and flavonols. High natural sugar content; consume mindfully in GDM. ^4^ Caution: Contains high levels of oxalic acid, which may interfere with calcium absorption and mineral balance during gestation. ^5^ Caution: Fennel in culinary amounts is safe, but concentrated supplements should be avoided due to potential estrogenic effects. ^6^ Caution: Culinary use (sprinkling on food) is safe. High-dose medicinal supplementation is contraindicated due to risk of uterine stimulation and emmenagogue properties. ^7–10^ Rich in catechins, but contains caffeine (requires limit <200 mg/day), and may interfere with folate absorption if consumed in excess. ^12–14^ Consume mindfully due to caffeine and sugar.

**Table 2:** An update on in vivo and in vitro studies (research published in 2025 and 2026) regarding flavonoids and their effects in pre-eclampsia and gestational diabetes mellitus.

Disorder/Substances	In Vitro or In Vivo Model	Mode of Action	References
PRE-ECLAMPSIA			
Baicalin	Human umbilical vein endothelial cells (HUVECs) exposed to hydrogen peroxide	downregulating MAPK/ ERK1/2 expression reducing apoptosis restoring MPO	Cao et al. [39]
Kaempferol	an L-NAME-induced PE rat model and a cellular hypoxia/reoxygenation model	↓ maternal hypertension, ↓ proteinuria, ↓ fetal growth restriction, ↓ oxidative stress, restore autophagy flux, upregulation PINK1 and Parkin, promotion STAT3 phosphorylation and its mitochondrial translocation, suppression NLRP3 inflammasome activation and pyroptosis	Yang et al. [40]
Luteolin	Animal model	↓ MAP, UARI, sFlt-1, and ET-1 compared to control group No adverse maternal or fetal outcomes were observed	Eddy et al. [41]
Silibinin	an L-NAME-induced PE rat model	↓ TNF-α, IL-1β, IFN-γ ↓ Hsp70 levels in the placenta and liver homogenate ↓ activation of NF-κB	Bannwart-Castro et al. [42]
GDM			
Homoplantaginin	GDM rats	↑ bodyweight, ↑ survival index of pups, ↓ FBG ↓ inflammation and oxidative stress responses	Yu et al. [43]
Tectorigenin	STZ-induced GDM in pregnant rats	↓ blood glucose, HOMA-IR, free fatty acids, visfatin, lipid peroxidation, VLDL, TC, TG, LDL cholesterol, TNF-α, IL-1β, IL-6, TGF-β, MCP-1, ICAM-1, VCAM-1, NF-κB, COX-2, iNOS, TLR4, MyD88, and NLRP3 levels; ↑ serum *C*-peptide, hepatic glycogen, fetal weight, placental weight, placental index, SOD, GPx, CAT, GSH, TAC, HO-1, Nrf2, IL-10, and HDL cholesterol levels modulation of the HO-1/Nrf2 and NF-κB/COX-2/iNOS signaling pathways.	Guo et al. [44]
alpinumisoflavone	STZ-induced GDM in pregnant rats	↓ blood glucose, OGTT and ITT responses, HbA1c, resistin, visfatin, leptin, free fatty acid, lipid parameters, lipogenic gene expression, oxidative stress markers, inflammatory cytokines, COX-2, ICAM-1, VCAM-1, and the mRNA expression of NF-κB p65 ↑ plasma insulin, serum *C*-peptide, adiponectin, hepatic glycogen, maternal body weight, and fetal weight and development ↓ placental weight and placental index	Zhu et al. [45]
Icariin	GDM cell model	↓ high glucose-induced intracellular LDH level	Yang et al. [46]
baicalin	GDM rats	improve blood lipid metabolism and immune function inhibition the RhoA/ROCK pathway	Lu et al. [47]
Anthocyanin-rich bilberry (*Vaccinium myrtillus* L.) extract	GDM rats	↓ hyperglycemia, ↓ insulin resistance, ↓dyslipidemia, ↓ inflammation, and oxidative stress ↑ *Lactobacillus*	Jiang et al. [48]

↑—increase, ↓—decrease, CAT—catalase, COX-2—cyclooxygenase-2, EGFR—epidermal growth factor receptor, ER—endoplasmic reticulum, ERK1/2—extracellular signal-regulated kinase 1/2, ET-1—endothelin-1, FBG—fasting blood glucose, GDM—gestational diabetes mellitus, GPx/GPx4—glutathione peroxidase/glutathione peroxidase 4, GSH—reduced glutathione, HbA1c—glycated hemoglobin, HDL—high-density lipoprotein, HO-1—heme oxygenase-1, HOMA-β—homeostatic model assessment of beta-cell function, HOMA-IR—homeostatic model assessment of insulin resistance, HOMA-ISI—homeostatic model assessment of insulin sensitivity index, Hsp70—heat shock protein 70, HUVECs—human umbilical vein endothelial cells, ICAM-1—intercellular adhesion molecule 1, IFN-γ—interferon gamma, IL-1β/IL-6/IL-10—interleukin 1 beta/interleukin 6/interleukin 10, iNOS—inducible nitric oxide synthase, ITT—insulin tolerance test, IUGR—intrauterine growth restriction, LDH—lactate dehydrogenase, LDL—low-density lipoprotein, L-NAME—N(ω)-nitro-L-arginine methyl ester, MAP—mean arterial pressure, MAPK—mitogen-activated protein kinase, MCP-1—monocyte chemoattractant protein-1, MPO—myeloperoxidase, MyD88—myeloid differentiation primary response 88, NF-κB/NF-κB p65—nuclear factor kappa B/nuclear factor kappa B p65 subunit, NLRP3 —NOD-like receptor family pyrin domain containing 3, Nrf2—nuclear factor erythroid 2-related factor 2, OGTT—oral glucose tolerance test, Parkin—Parkin RBR E3 ubiquitin protein ligase, PE—pre-eclampsia, PI3K—phosphoinositide 3-kinase, PINK1—PTEN-induced kinase 1, RAGE—receptor for advanced glycation end products, RhoA/ROCK—Ras homolog family member A/Rho-associated protein kinase, SOD—superoxide dismutase, STAT3—signal transducer and activator of transcription 3, STZ—streptozotocin, TAC—total antioxidant capacity, TC—total cholesterol, TG —triglycerides, TNF-α—tumor necrosis factor alpha, UARI—uterine artery resistance index, VCAM-1—vascular cell adhesion molecule 1, VLDL—very low-density lipoprotein.

**Table 3:** Overview of emerging research directions on the protective effects of flavonoids against infectious, parasitic, and environmental gestational complications.

Target Condition	Flavonoid/Extract	Study Model	Key Biological Outcome	References
Embryonic loss *Listeria monocytogenes*	Baicalin	Pregnant BALB/c mice and macrophages	Alleviates LM-induced embryonic loss and pyroptosis	Zhang et al. [49]
Congenital toxoplasmosis *Toxoplasma gondii*	Quercetin	Human placental villous explants, cell models and in silico docking	Irreversibly inhibits parasite adhesion, invasion, and proliferation in placental explants; reduces lipid droplets, restores redox balance, modulates cytokines (IL-4, IL-6, IL-8), and targets TgHGPRT.	Souto et al. [50]
Congenital Zika Syndrome/Microcephaly Zika virus (ZIKV)	Naringenin	Human placental villous explants and pregnant mouse model	Exerts dual antiviral and anti-inflammatory action at the placental barrier; suppresses LPS-exacerbated ZIKV replication and alleviates microcephaly-related brain structural malformations in neonates	Branco et al. [51]
Prenatal nephrotoxicity/Fibrosis Methylmercury (MeHg)	Fisetin	Pregnant Wistar rats and offspring	Mitigates gestational MeHg-induced nephrotoxicity in offspring; normalizes renal biomarkers (BUN, creatinine), downregulates fibrotic markers, and preserves kidney tissue architecture	Shafi et al. [52]
ZnO NP-induced placental toxicity	Quercetin	Pregnant rat model	Attenuates ZnO NP-induced placental toxicity and tissue damage by mitigating oxidative stress, suppressing ER stress pathways and apoptosis	Alshaikh et al. [53]
Autism spectrum disorder	Chrysin	Prenatally VPA-exposed male Wistar rats	Ameliorates autistic-like behaviors (social deficits, anxiety, hyperalgesia)	Adeniyi et al. [54]
Neuroinflammation and memory impairment	Cherry juice (rich in anthocyanins)	Rat offspring exposed to LPS during gestation or gestation–lactation	Exerts neuroprotective effects against maternal immune activation; improves short- and long-term memory, alleviates anxiety-like behavior, normalizes systemic leukocyte levels, and restores hippocampal cell counts	Virgen-Gen et al. [55]
Recurrent spontaneous abortion	Quercetin	Injured HTR-8/SVneo trophoblasts (in vitro) & molecular docking	Alleviates trophoblast dysfunction by improving viability, restoring cell migration, inhibiting apoptosis, and maintaining redox homeostasis	Liu et al. [56]
Recurrent pregnancy loss (RPL)	Baicalein	Pregnant RPL mouse model	Attenuates fetal loss and placental injury by inhibiting ferroptosis, lipid peroxidation, and iron accumulation	Li et al. [57]
Gestational/Periparturient oxidative stress	Natural antioxidant blend (tocopherol + rosemary extract rich in flavonoids	Pregnant Beetal goats and offspring	Enhances maternal antioxidant defense, improves colostrum and milk biochemical composition, and increases neonatal birth weight	Zaib et al. [58]

BUN—blood urea nitrogen, ER—endoplasmic reticulum, HTR-8/SVneo—human extravillous trophoblast cell line, IL—interleukin (IL-4, IL-6, IL-8), LM—*Listeria monocytogenes*, LPS—lipopolysaccharide, MeHg—methylmercury, RPL—recurrent pregnancy loss, TgHGPRT—*Toxoplasma gondii* hypoxanthine-guanine phosphoribosyltransferase, VPA—valproic acid, ZIKV—Zika virus, ZnO NPs—zinc oxide nanoparticles.

## Data Availability

No new data were created or analyzed in this study. Data sharing is not applicable to this article.

## Supplementary Materials

The following supporting information can be downloaded at https://www.mdpi.com/article/10.3390/nu18213482/s1, Table S1. Summary of (A) clinical and prospective studies and (B) meta-analyses on flavonoid intake regarding maternal and neonatal outcomes.
