# Supplementary material for: Clinical Applications of Flavonoids in Pregnancy: Potential Therapeutic Strategies for Gestational Disorders—An Update
Source: Nutrients. 2026 Oct 23;18(21):3482. doi: 10.3390/nu18213482 (PMC13765491; doi:10.3390/nu18213482)
Supplement: Supplementary file 1 [file nutrients-18-03482-s001.zip › nutrients-4647211-supplementary.pdf]

nutrients

MDPI

Supplementary Table S1. Summary of (A) clinical and prospective studies and (B) meta-analyses on flavonoid intake regarding maternal and neonatal outcomes.

Study (year)	Study Design and Comparator (if applicable)	Number of participants	Drug/Substance	Dosage [mg/d]	Condition	Treatment/ use duration	Endpoints/Measures	Outcomes	Limitations
**A. Clinical and prospective studies regarding maternal and neonatal outcomes.**									
**PRE-CONCEPTION & FERTILITY OUTCOMES**									
Vaez (2023) [1]	Randomized controlled trial (RCT)	72 women with PCOS (undergoing IVF)	Quercetin	500 mg/day (oral)	Polycystic Ovary Syndrome (PCOS), oxidative stress, and infertility (IVF protocol)	40 days (from the start of the menstrual cycle until the day of ovulation)	• Inflammatory and Hormonal markers<br>• Clinical / Fertility Outcomes: Oocyte grade, embryo grade before IVF, and pregnancy rate.	• Improvement in oocyte grade and embryo grade before IVF<br>• Significant increase in pregnancy rate in PCOS patients<br>• Significant decrease in LH levels (4.35 ± 1.62 to 3.06 ± 1.43, p = 0.029)<br>• Significant reduction in TNF-α (p = 0.008) and IL-6 (p = 0.001) in the quercetin group.	Absence of a standard hormone-only treatment control group and lack of visceral adiposity quantification.
**GESTATIONAL DISORDERS & COMPLICATIONS**									
Soldavini (2022) [2]	Double-blind randomized controlled trial	40 women with gestational diabetes (GDM) at 24–28 weeks of gestation	Anthocyanins, Omega-3 fatty acids, and alpha-cyclodextrins vs.	Omega-3 (2.4 g/d at breakfast), Anthocyanins (108 mg/d in 3 divided	Gestational Diabetes Mellitus (GDM), metabolic/inflammatory alterations,	12 weeks	• Blood and urine measurements of metabolic, inflammatory, and antioxidant parameters	• Omega-3 and antioxidant supplementation produced no statistically significant changes in metabolic or inflammatory parameters compared	Small sample size

			dietary counseling alone	doses), and Alpha-cyclodextrins (15 g/d in 3 divided doses)	and prediction of need for pharmacological hypoglycemic therapy		• Requirement for pharmacological hypoglycemic treatment	with strict, individualized (1-on-1) dietary counseling alone	
Jaworsky (2023) [3]	randomized controlled trial	38 women with GDM (Nutrition education: n=18; Nutrition intervention: n=20)	Whole berries & leafy vegetables (+ postprandial walking exercise)	1 cup of whole berries + 1 cup of leafy vegetables daily	Gestational Diabetes Mellitus (GDM) and cardiometabolic risks	12 weeks	• Blood samples, anthropometric measures, dietary and physical activity data (daily food and activity logs) collected at baseline and week 12	• Significantly improved random blood glucose, HDL cholesterol, serum antioxidant capacity, and fiber/antioxidant intake<br>• reduced serum IL-6 ($p < 0.05$)	Inability to isolate independent effects of berry intake, leafy vegetables, or postprandial exercise
Kojima (2026) [4]	Prospective birth cohort study	93,759 pregnant women with singleton pregnancies (2,096 GDM cases, 2.2%)	Total fruit, flavonoid-rich fruits, non-flavonoid-rich fruits, and 100% fruit juice.	assessed as dietary intake via FFQ	Gestational Diabetes Mellitus (GDM)	Dietary intake during the past year assessed in the first trimester	• Effect of flavonoid intake in the first trimester on the GDM risk	• Inversely associated with GDM risk for total fruit (aOR 0.82), flavonoid-rich fruits (aOR 0.80), and 100% juice (aOR 0.91 per 100 mL/d), but not non-flavonoid fruits (NS).	Potential residual or unmeasured confounding due to co-consumption of other polyphenol-rich foods (coffee, soy, chocolate)
Liu (2026) [5]	Prospective birth cohort study	2,160 pregnant women	Dietary polyphenols (total polyphenols, flavonoids,	assessed as dietary intake via FFQ	Patient with gestational hypertension (GH)	Dietary intake during early pregnancy	• GH<br>• Food Frequency Questionnaire (FFQ)	• Higher maternal polyphenol and flavonoid intake (especially from fruits) was significantly	dietary intake was evaluated in early

			phenolic acids; primarily fruit-derived)					associated with lower GH risk, with effects plateauing at moderate-to-high doses.<br>• Systemic immune-inflammation index partially mediated the protective association (8.19% for total polyphenols; 8.04% for flavonoids).	pregnancy only
Mei (2025) [6]	Observational twin cohort study.	143 twin families (12-month-old infants).	Catechin (fecal metabolite/flavonoid and phenylpropanoid biosynthesis)	n/a	Preterm birth, chorionicity, gut microbiota dysbiosis, and physical/neurobehavioral development in twins	Evaluation at 12 months of age	• 16S rRNA sequencing (gut microbiota diversity and genera).<br>• Untargeted metabolomics (fecal metabolites)<br>• Statistical models<br>• Physical and neurobehavioral development assessments	• Preterm birth shapes 12-mo twin gut microbiota more than genetics; 6 genera and 18 metabolites (including catechin via flavonoid pathways) correlate with physical and neurobehavioral development.	predominant C-section delivery, lack of neonatal antibiotic data
Zhang (2025) [7]	Cohort study derived from a community-based randomized controlled trial	1 trial/cohort (2,244 pregnant women; 234 cases of SGA)	Dietary anthocyanidins (total anthocyanidins and subclasses: cyanidin, peonidin)	Median intake: 28.7 mg/d (calculated via validated FFQ)	Small-for-gestational-age (SGA) infants and dietary inflammatory potential during pregnancy	Follow-up during pregnancy (up to 39.7 gestational weeks)	• SGA incidence (hospital records)<br>• Anthocyanidin intake (FFQ)<br>• Energy-adjusted dietary inflammatory index	• Anthocyanidin intake (total, cyanidin, peonidin) significantly reduced SGA risk (OR = 0.96), with stronger protection observed among women with high dietary inflammatory	Single-country cohort (China), limiting generalizability to other populations

								potential (highest EDII tertile: OR = 0.67; β = −0.40)	
				**POSTPARTUM & PERIOPERATIVE CARE**					
Astaneh (2026) [8]	triple-blind, three-arm randomized controlled trial	102 multiparous women	Elaeagnus angustifolia extract capsules rich in flavonoids	500-mg capsule	postpartum afterpains	Two hours after vaginal delivery and continuing for 24 h, participants received one 500-mg capsule every six hours	• mean pain intensity score,<br>• emotional pain quality score,<br>• physical pain quality score,<br>• pain quality score	• reduction in the severity and quality of postpartum afterpains	relatively short follow-up period (24 h)<br><br>Potential capsule preparation bias
Elmokadem (2025) [9]	Prospective, double-blinded, randomized controlled trial (RCT)	80 patients undergoing elective cesarean section under spinal anesthesia	Quercetin (oral)	500 mg	Acute postoperative pain following elective cesarean section	single oral dose administered 1 hour before surgery	• Postoperative pain intensity via 10 cm Visual Analog Scale (VAS) at 2, 6, 12, and 24 hours<br>• Time to first rescue analgesic request, total morphine consumption, PONV incidence, time to physical activity initiation, functional activity, patient satisfaction (POD2), hospital length of stay, and adverse effects.	• Preoperative quercetin reduced VAS pain (2–24 h), delayed rescue analgesia (3.9 vs 2.7 h), accelerated mobilization (15.2 vs 19.0 h), and improved satisfaction without affecting morphine consumption, PONV, or adverse events (NS)	Limited by a single-dose regimen (500 mg), a small sample size

**OFFSPRING & PEDIATRIC HEALTH OUTCOMES**									
Yang (2026) [10]	Prospective birth cohort study	86,012 mother–child pairs (children aged ≤1 year); 75,307 mother–child pairs (children aged 1–3 years).	Isoflavones (total daidzein and genistein from dietary intake)	assessed as dietary intake via FFQ	Infant allergy	Maternal dietary consumption during pregnancy	• Physician-diagnosed food allergy in children	• Protective association observed across all ages in female children, but only at 1–3 years in male children	the risk of underreporting isoflavone consumption
Dawson (2026) [11]	Prospective prebirth cohort study.	599 mother–infant dyads	Dietary proanthocyanidins (polyphenols), maternal urinary hippuric acid (polyphenol metabolite), and gut commensal *Prevotella copri*	assessed as dietary intake via FFQ	Infant food allergy at 1 year of age.	Maternal dietary data, fecal and urine sampling in the 3rd trimester; infant follow-up at 12 months	• Prenatal urinary hippuric acid (NMR),<br>• proanthocyanidin intake,<br>• fecal *P. copri*	• Higher maternal proanthocyanidin intake and urinary hippuric acid (increased by *P. copri*) significantly reduced infant food allergy risk (OR = 0.58 and OR = 0.62, respectively)	single urine measurement in 3rd trimester
**B. Meta-analyses on flavonoid intake regarding maternal and neonatal outcomes.**									
**Study (year)**	**Study Design and Comparator**	**Number of trials (subjects)**	**Drug/Substance**	**Dosage [mg/d]**	**Condition**	**Treatment/ use duration**	**Endpoints/Measures**	**Outcomes**	**Limitations**
Zhong (2025) [12]	Meta-analysis of RCTs combined with	8 RCTs (1,563	Probiotics (specifically *Bifidobacterium* +	n/a	Overweight or obese pregnant women;	n/a	• Risk of adverse pregnancy outcomes	• No statistically significant overall difference between probiotics and control	

	network pharmacology and molecular docking	pregnant women)	*Lactobacillus* combinations; bioactive metabolites: quercetin, kaempferol, caffeic acid, equol, doconexent, arctigenin, etc.)		adverse pregnancy outcomes (specifically risk of SGA - small-for-gestational-age)			for general adverse pregnancy outcomes	
Nguyen (2025) [13]	meta-analysis of RCTs	14 RCTs evaluating 6 polyphenol candidates (EGCG trial: n=349; Resveratrol trial: n=349)	Polyphenolic products: Epigallocatechin gallate (EGCG), Resveratrol, *Salvia miltiorrhiza*, *Bryophyllum pinnatum*, raspberry extract, cranberry extract	n/a	Preeclampsia (preeclampsia-related maternal and neonatal outcomes)	n/a	• Clinical outcomes combining the core outcome set for preeclampsia and WHO priority outcomes (e.g., time to BP control, time to next hypertensive crisis)	• Addition of the flavonoid EGCG or resveratrol to nifedipine significantly reduced time to blood pressure control (MD = -14.10 min and -15.50 min, respectively) and extended time to next hypertensive crisis (MD = +3.10 h and +2.50 h), whereas other polyphenol candidates showed no significant differences.	Limited sample sizes caused non-informative meta-analytic effects

AA/EPA— arachidonic acid to eicosapentaenoic acid ratio, AKT1— AKT serine/threonine kinase 1, aOR— adjusted odds ratio, BCL2— B-cell lymphoma 2, BMI— body mass index, BP— blood pressure, CCND1— cyclin D1, CI— confidence interval, CRP— C-reactive protein, DM— diabetes mellitus, EDII— energy-adjusted dietary inflammatory index, EGCG— epigallocatechin gallate, EGFR— epidermal growth factor receptor, ESR1— estrogen receptor 1, FFQ— food frequency questionnaire, GD— gestational diabetes, GDM— gestational diabetes mellitus, GEE— generalized estimating equations, GH— gestational hypertension, HDL / HDL-C— high-density lipoprotein cholesterol, HSP90AA1— heat shock protein 90 alpha family class A member 1, IL-6— interleukin 6, IVF— in vitro fertilization, LH— luteinizing hormone, MD— mean difference, MTOR— mechanistic target of rapamycin kinase, n/a— not available or not applicable, NMR— nuclear magnetic resonance, NS— non-significant, OMIM— Online Mendelian Inheritance in Man, OR— odds ratio, PAF— platelet-activating factor, PCOS— polycystic ovary syndrome, POD2— postoperative day 2, PONV— postoperative nausea and vomiting, Pre-DM— pre-diabetes mellitus, PROSPERO— International Prospective Register of Systematic Reviews, RCT— randomized controlled trial, ROBINS-E— Risk of

Bias In Non-Randomized Studies–of Exposure, RR— risk ratio, 16S rRNA— 16S ribosomal RNA, SGA— small-for-gestational-age, SRC— SRC proto-oncogene, non-receptor tyrosine kinase, STAT3— signal transducer and activator of transcription 3, T1DM— type 1 diabetes mellitus, T2DM— type 2 diabetes mellitus, TNF-α— tumor necrosis factor-alpha, TP53— tumor protein p53, USDA— United States Department of Agriculture, VAS— visual analog scale, WHO— World Health Organization

1. Vaez, S.; Parivr, K.; Amidi, F.; Rudbari, N.H.; Moini, A.; Amini, N. Quercetin and polycystic ovary syndrome; inflammation, hormonal parameters and pregnancy outcome: A randomized clinical trial. *Am J Reprod Immunol* **2023**, *89*, e13644, doi:10.1111/aji.13644.
2. Soldavini, C.M.; Piuri, G.; Rossi, G.; Corsetto, P.A.; Benzoni, L.; Maggi, V.; Privitera, G.; Spadafranca, A.; Rizzo, A.M.; Ferrazzi, E. Maternal AA/EPA Ratio and Triglycerides as Potential Biomarkers of Patients at Major Risk for Pharmacological Therapy in Gestational Diabetes. *Nutrients* **2022**, *14*, doi:10.3390/nu14122502.
3. Jaworsky, K.; DeVillez, P.; Alexander, J.M.; Basu, A. Effects of an Eating Pattern Including Colorful Fruits and Vegetables on Management of Gestational Diabetes: A Randomized Controlled Trial. *Nutrients* **2023**, *15*, doi:10.3390/nu15163624.
4. Kojima, A.; Ikehara, S.; Miyazaki, J.; Kawasaki, R.; Iso, H. Association between Total Fruit Consumption and Risk of Gestational Diabetes Mellitus: The Japan Environment and Children's Study. *Ann Nutr Metab* **2026**, *82*, 81-89, doi:10.1159/000549407.
5. Liu, H.; Yan, K.; Chen, Z.; Cao, X.; Mu, Y.; Lu, X.; Ran, M.; Chen, G.; Hao, L. Maternal Polyphenol Intake and Gestational Hypertension: Nonlinear Associations and Inflammatory Mediation. *Mol Nutr Food Res* **2026**, *70*, e70440, doi:10.1002/mnfr.70440.
6. Mei, H.; Hu, L.; Yang, M.; Xiang, F.; Zheng, H.; Cai, X.; Hou, G.; Li, R.; Peng, A.; Zhang, J., et al. Preterm birth alters the gut microbiota, metabolome and health outcomes of twins at 12 months of age. *Front Cell Infect Microbiol* **2025**, *15*, 1700965, doi:10.3389/fcimb.2025.1700965.
7. Zhang, B.; Xu, K.; Mi, B.; Yan, H.; Wang, D.; Dang, S.; Men, K. Maternal Dietary Anthocyanidin, Dietary Inflammatory Potential, and Risk of Small-for-Gestational-Age in China. *Nutrients* **2025**, *17*, doi:10.3390/nu17203187.
8. Zabeti Asnad Astaneh, S.P.; Karimi, F.Z.; Salar, R.; Mazloum, S.R. The impact of Elaeagnus angustifolia fruit extract capsule on postpartum afterpains in multiparous women: a randomized, triple-blind, placebo-controlled clinical trial. *Explore (NY)* **2026**, *22*, 103464, doi:10.1016/j.explore.2026.103464.
9. Mohamed Elmokadem, E.; Khaled Abou El Fadl, D.; Bassiouny, A.M.; Mahmoud, M.; Samy, M.; El Said, N.O. The Adjunctive Effect of Quercetin on Postoperative Pain Management Following Cesarean Section: A Randomized Controlled Study. *Drug Des Devel Ther* **2025**, *19*, 6009-6024, doi:10.2147/dddt.S526188.
10. Yang, G.; Hisada, A.; Yamamoto, M.; Takatani, R.; Konno, Y.; Shimojo, N.; Sakurai, K. Association between maternal dietary isoflavone consumption during pregnancy and childhood food allergy in offspring: The Japan Environment and Children's study. *Pediatr Allergy Immunol* **2026**, *37*, e70303, doi:10.1111/pai.70303.

11. Dawson, S.L.; Collier, F.; O'Hely, M.; Holmes, E.; Holland, L.; Morgan, R.; Lodge, S.; Gray, N.; Phipps, S.; Burgner, D., et al. Maternal prenatal urinary metabolites associate with infant food allergy. *Pediatr Allergy Immunol* **2025**, *36*, e70252, doi:10.1111/pai.70252.
12. Zhong, T.; Sun, J.; Miao, M.; Sun, G. The mechanism of probiotics in pregnancy outcomes in overweight or obese pregnant women based on meta-analysis, network pharmacology and molecular docking. *BMC Pregnancy Childbirth* **2025**, *25*, 886, doi:10.1186/s12884-025-07999-x.
13. Nguyen, P.Y.; Sanderson, B.; Makama, M.; Mills, K.; Ammerdorffer, A.; Gülmezoglu, A.M.; Vogel, J.P.; McDougall, A.R.A. Polyphenols for the Prevention or Management of Preeclampsia: A Systematic Review and Meta-Analysis. *Bjog* **2025**, *132*, 867-879, doi:10.1111/1471-0528.18106.
